# Prevalence of placenta previa among deliveries in Mainland China: A PRISMA-compliant systematic review and meta-analysis

**DOI:** 10.1097/MD.0000000000005107

**Published:** 2016-10-07

**Authors:** Dazhi Fan, Song Wu, Wen Wang, Lihong Xin, Guo Tian, Li Liu, Jinping Feng, Xiaoling Guo, Zhengping Liu

**Affiliations:** a Department of Obstetrics, South Medical University Affiliated Maternal & Child Health Hospital of Foshan, Foshan, Guangdong; b School of Integrated Traditional and Western Medicine, Anhui University of Chinese Medicine, Hefei, Anhui; c Department of Epidemiology and Biostatistics, School of Public Health, Anhui Medical University, Hefei, Anhui; d The First Affiliated Hospital, College of Medicine, Zhejiang University, Hangzhou, Zhejiang, China.

**Keywords:** Mainland China, meta-analysis, placenta previa, prevalence

## Abstract

**Background:**

Placenta previa is characterized by the abnormal placenta overlying the endocervical os, and it is known as one of the most feared adverse maternal and fetal-neonatal complications in obstetrics.

**Objectives:**

We aimed to obtain overall and regional estimates of placenta previa prevalence among deliveries in Mainland China.

**Methods:**

The research was performed a systematic review, following the Meta-analysis of observational studies in epidemiology (MOOSE) guidelines for systematic reviews of observational studies, and the preferred reporting items for systematic reviews and meta-analysis (PRISMA) statement for reporting systematic reviews and meta-analysis. Electronic databases were searched and included hospital-based studies that reported placenta previa prevalence in Mainland China. Random-effects meta-analyses were used to pool prevalence estimates of placenta previa. Meta-regression analyses were performed to explore sources of heterogeneity across the included studies. For exploring the geographical distributions of placenta previa, the ArcGIS software (Esri) was used to construct the map of prevalence.

**Results:**

A total of 80 articles and 86 datasets (including 1,298,548 subjects and 14,199 placenta previa cases) from 1965 through 2015 were included. The pooled overall prevalence of placenta previa among deliveries was 1.24% (95% confidence interval [CI], 1.12–1.36) in Mainland China during 1965 to 2015. And, the trend in the prevalence of placenta previa was steady. The occurrence rate of placenta previa in the region groups Northeast, North, Northwest, Central China, East, South, and Southwest was 1.20%, 1.01%, 1.10%, 1.15%, 0.93%, 1.42%, and 2.01%, respectively. The prevalence map based on a geographic information system showed an unequal geographic distribution.

**Conclusions:**

The results showed that placenta previa is currently a high-burden disease in Mainland China. This review would be useful for the design of placenta previa planning and implementation adequate health care systems and treatment programs in Mainland China.

## Abstract 2

Supplemental Digital Content is available in the text

## 1 Introduction

Placenta previa (PP) is characterized by the abnormal placenta overlying the endocervical os, and it is known as one of the most feared adverse maternal and fetal-neonatal complications in obstetrics.^[1]^ Women with placenta previa are at an approximately 4-fold increased risk of second trimester vaginal bleeding.^[2]^ In addition, peripartum hysterectomy, blood transfusion, postpartum hemorrhage, and placenta accreta are also associated with placenta previa. Fetal complications with placenta previa are primarily those prone for prematurity and intrauterine growth restriction. In turn, neonatal mortality rates are increased by about 4-fold in singleton pregnancies with placenta previa.^[3]^ Although the etiology of placenta previa is still unknown, the pathogenesis is likely to be the result of endometrial damage and uterine scarring.^[4]^

A study reported that the overall prevalence of placenta previa was approximately 5 per 1000 pregnancies by world region, however, there is also some evidence suggestive of regional variation.^[5]^ Owing to several factors such as the increasing rates of caesarean section, ongoing nutritional transition, and increasing rates of advanced maternal age, the prevalence of placenta previa seems to be rising trend in recent years in Mainland China. The prevalence of placenta previa in China (2.01%) was first reported by Guo et al^[6]^ in 1965 with 220 cases in 10,919 pregnancies. However, another study^[7]^ in 2015 noted that the prevalence of placenta previa was 1.14%, nearly 1 of 2 times lower than the level reported half a century before. Since the 1980s, most surveys on placenta previa have been conducted in different parts of Mainland China. The prevalence reports varied considerably, ranging from 0.24% in Beijing^[8]^ to 5% in Hainan.^[9]^ These differences in prevalence rates may be due to differences among the studies in regional variation, survey time, and maternal age.

Although previously studies provided lots of valuable information, they focused on one or several provinces rather than nationally representative sample of pregnancies population. The data of placenta previa remain incompletely limited. There has been no meta-analysis pooling the prevalence of placenta previa attempts across different provinces to date. Therefore, we aimed to fill this gap in the evidence by providing overall and regional estimates of the overall pooled prevalence of placenta previa from previously survey and estimate a comprehensive picture of placenta previa in Mainland China.

## 2 Methods

The prevalence research was performed a systematic review, in accordance to the Meta-analysis of observational studies in epidemiology (MOOSE) guidelines for systematic reviews of observational studies (Supplementary Table 1).^[10]^ In addition, we also conducted in line with the preferred reporting items for systematic reviews and meta-analysis (PRISMA) statement for reporting systematic reviews and meta-analysis (Supplementary Table 2).^[11]^

### 2.1 Search strategy and selection criteria

Three English (PubMed, Cochrane Library, and Elsevier Science Direct) and two Chinese (the Chinese Biological Medical Literature database [CBM] and the Chinese National Knowledge Infrastructure database [CNKI]) databases for studies containing the data were searched using the following search terms: “placenta previa,” “PP,” “prevalence,” “epidemiology,” “survey,” and “China” from the established date up to July 2015; and the search was later updated in January 2016. We also manually checked the relevant eligible literatures through cross-references of identified in the reference lists within both original and review articles.

The studies were included if they met the following inclusion criteria: studies described the prevalence of placenta previa among deliveries or either provided the number of cases of placenta previa and the total number of deliveries or births or sufficient data for calculating the prevalence; studies were reported in Mainland China; because of cultural differences from Mainland China, articles from Hong Kong, Macao, and Taiwan were excluded; the full-text articles written in English or Chinese.

### 2.2 Data extraction

After initial evaluation, two authors (DF and SW) independently and carefully evaluated the articles and performed the data extraction according to standard selection criteria in Microsoft Excel. The included items were: first author, years published, enrolment period, location, maternal age, sample size of placenta previa, and the total deliveries. When disagreements existed between the two authors, discussion was performed or via consultation with another reviewer (GT). If necessary, the first or corresponding author of the published study was also contacted to provide relevant information for our analysis.

### 2.3 Methodological quality assessment

To appraise the risk of bias, the methodological quality of included studies was assessed by two independent authors (WW and LL) defined as adherence to the reporting of observational studies in epidemiology (STROBE) guideline^[12]^ which was adopted in previous meta-analysis.^[13]^ The STROBE included five core components including sample population, sample size, participation rate, outcome assessment, and analytical methods to control for bias. Briefly, the item is assessed by scoring (low risk = 2, moderate risk = 1, high risk and unclear = 0) each bias type for each publication and the total score is used as the summary assessment of risk of bias. When there was a disagreement in the evaluation of a study between the authors, it was solved by consensus of the whole team.

### 2.4 Ethical Approval

Being a systematic review of published literature, no ethical approval was needed for this manuscript.

### 2.5 Statistical analysis

The main outcome in this system review was the prevalence of placenta previa among deliveries with a 95% confidence interval (95% CI). For pooled data, the *I*^2^ statistic was used to estimate statistical heterogeneity. The result was interpreted as low, moderate, or high levels of heterogeneity, and found high levels (*I*^2^ > 50%) heterogeneity among the study findings. Because of heterogeneity, the DerSimonian–Laird random-effects model meta-analysis^[14]^ was chosen to calculate pooled prevalence and 95% CI. To explore potential sources of heterogeneity, subgroup analyses and meta-regression of the prevalence were carried out. Subgroups were divided by location, maternal age, survey year, quality score, and hospital level. Potential publication bias was explored using a funnel plot (prevalence vs. standard error) and Egger test. Statistical analyses were conducted using the STATA 11.0 (Stata-Corp, College Station, TX). And *P* ≤ 0.05 indicated the presence of statistically significant.

For describing the geographical distributions of the prevalence of placenta previa, the ArcGIS software version 10 system (the URL link: http://www.esri.com/) was used to construct the map: the pooled prevalence in each province was calculated by meta-analysis, respectively; the data of each pooled prevalence were then imported into ArcGIS for analysis.

## 3 Results

### 3.1 Search results and characteristics

**Figure 1:**
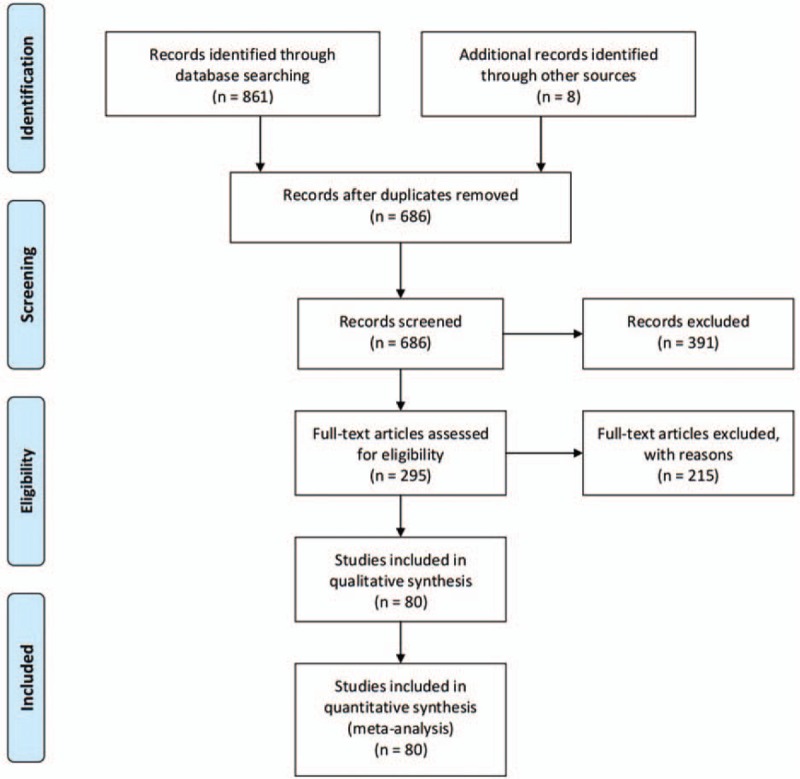
PRISMA flowchart showing the study selection process. PRISMA = preferred reporting items for systematic reviews and meta-analysis.

Figure 1 showed the study selection flow. The electronic database searches initially yielded 861 studies: PubMed (n = 116), Science Direct (n = 260), Cochrane Library (n = 0), CBM (n = 184), and CNKI (n = 301), and 8 additional records identified through other sources. One hundred and eighty-three (183) duplicate records were removed among the different databases. After initial screening the titles and abstracts, a total of 295 potentially eligible studies were selected for full-text review. Two hundred and fifteen (215) studies were excluded for not meeting the selection criteria. Finally, a total of 80 eligible retrospective cohort studies were included in our meta-analysis (Fig. 1).

All of the included studies were published from 1965 to 2015 in 25 provinces of the seven regions (Northeast: Liaoning, Heilongjiang; North: Beijing, Tianjin, Shanxi, Inner Mongolia; Northwest: Shaanxi, Ningxia, Xinjiang, Qinghai, Gansu; Central China: Hunan, Hubei, Henan; East: Shandong, Jiangsu, Zhejiang, Shanghai; South: Fujian, Guangdong, Guangxi, Hainan; Southwest: Sichuan, Yunnan, Guizhou). The prevalence of placenta previa in the selected 80 studies ranged from 0.24% to 5.00%. The lowest prevalence of placenta previa was found in Beijing in 1994, whereas the highest prevalence was observed in Hainan in 1997. Three, 4, 10, 25, 29, and 9 studies had a quality score of 9, 8, 7, 6, 5, and 4, respectively; these results show that in general (with a mean of 6), the studies were of acceptable quality. The characteristics of the 80 studies and 86 datasets included are shown in Supplementary Table 3.

### 3.2 Overall prevalence of placenta previa

As shown in Table 1, 80 studies and 86 datasets, which included a total sample size of 1,298,548 and 14,199 placenta previa cases, evaluated the prevalence of placenta previa in Mainland China. The overall prevalence of placenta previa was 1.24% (95% CI, 1.12–1.36), and the forest plot for the overall estimates was shown in Supplementary Fig. 1.

**Table 1:**
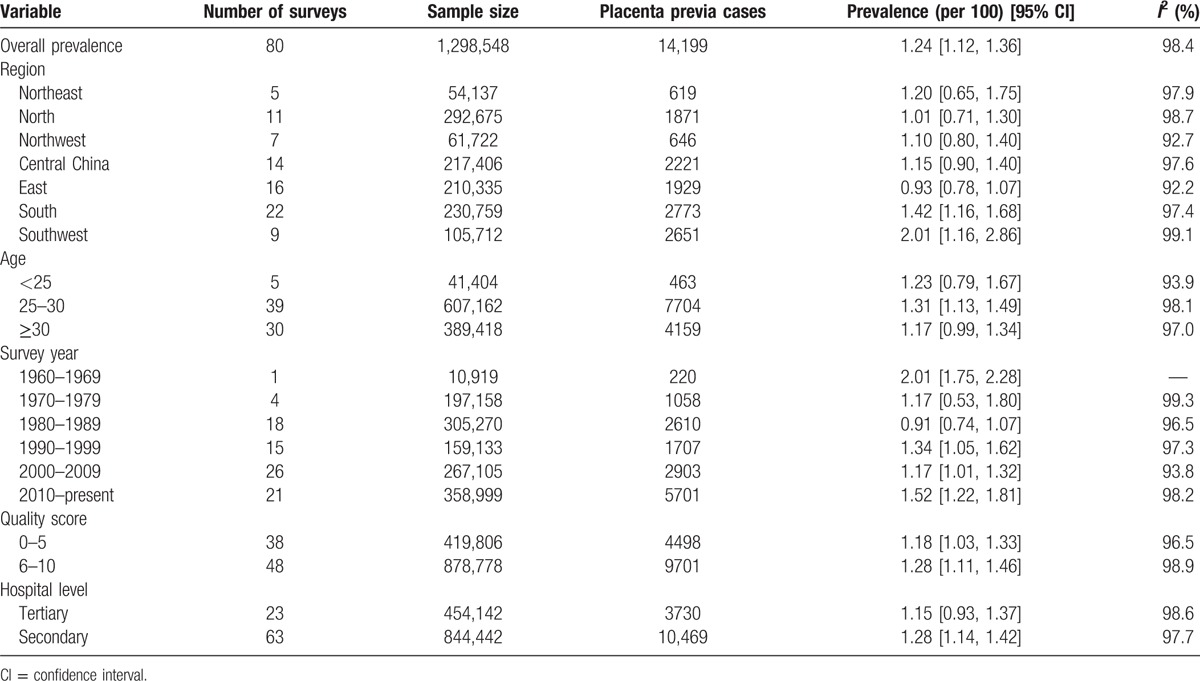
Prevalence of placenta previa in Mainland China and subgroup analysis.

### 3.3 Pooled prevalence by regions

The number of studies evaluating the region groups of Northeast, North, Northwest, Central China, East, South, and Southwest were 5, 11, 7, 14, 16, 22, and 9, respectively. The prevalence of placenta previa of Northeast, North, Northwest, Central China, East, South, and Southwest were 1.20% (95% CI, 0.65–1.75), 1.01% (95% CI, 0.71–1.30), 1.10% (95% CI, 0.80–1.40), 1.15% (95% CI, 0.90–1.40), 0.93% (95% CI, 0.78–1.07), 1.42% (95% CI, 1.16–1.68), and 2.01% (95% CI, 1.16–2.86), respectively (Table 1). The forest plot was shown in Supplementary Fig. 2.

### 3.4 Pooled prevalence by age

Five datasets provided the prevalence of placenta previa in aged <25 years, with a prevalence rate of 1.23% (95% CI, 0.79–1.67). Thirty-nine datasets evaluated the prevalence of placenta previa in aged 25 to 30 years, with an estimate of 1.31% (95% CI, 1.13–1.49). And, 30 datasets provided the prevalence of placenta previa in aged ≥30 years, with a prevalence rate of 1.17% (95% CI, 0.99–1.34) (Table 1). Supplementary Fig. 3 showed the forest plot.

### 3.5 Subgroup analysis based on survey year, quality score, and hospital level

As shown in Table 1, the prevalence of placenta previa in the survey year groups of 1960 to 1969, 1970 to 1979, 1980 to 1989, 1990 to 1999, 2000 to 2009, and 2010 to present was 2.01% (95% CI, 1.75–2.28), 1.17% (95% CI, 0.53–1.80), 0.91% (95% CI, 0.74–1.07), 1.34% (95% CI, 1.05–1.62), 1.17% (95% CI, 1.01–1.32), and 1.52% (95% CI, 1.22–1.81), respectively (Supplementary Fig. 4); the prevalence of placenta previa in the groups of 0 to 5 and 6 to 10 was 1.18% (95% CI, 1.03–1.33) and 1.28% (95% CI, 1.11–1.46), respectively (Supplementary Fig. 5); the prevalence of placenta previa in the hospital level of tertiary and secondary was 1.15% (95% CI, 0.93–1.37) and 1.28% (95% CI, 1.14–1.42), respectively (Supplementary Fig. 6).

### 3.6 Meta-regression and publication bias

To better explore the possible sources of heterogeneity among studies, a meta-regression analysis was performed. Region, mean age, publication year, quality score, and hospital level, which may be potential sources of heterogeneity, were tested by a meta-regression method. Through the regression model, we did not find a significant heterogeneity for the 5 variables listed above (Table 2). Funnel plots and Egger test were performed to assess the publication bias of the study. The shape of the funnel plots showed asymmetry, and the Egger test resulted in *P* < 0.001, which indicates that publication bias was existence.

**Table 2:**
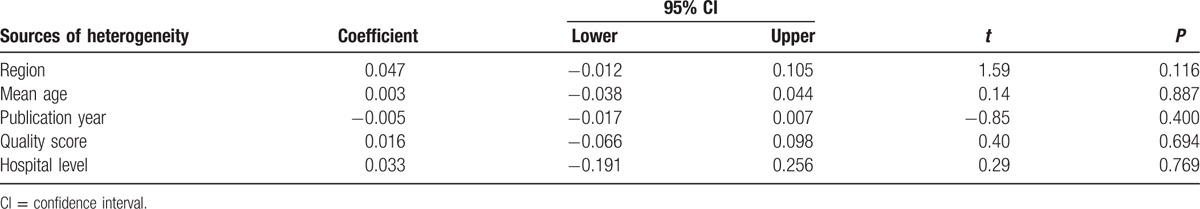
Meta-regression analysis on the included studies.

### 3.7 Trends in the prevalence of placenta previa

**Figure 2:**
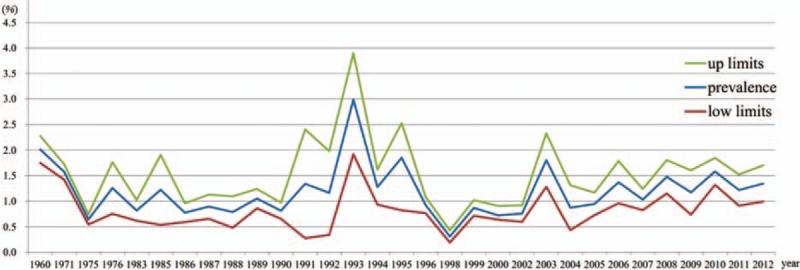
Pooled estimated prevalence of placenta previa in Mainland China with corresponding 95% confidence intervals from different survey year.

Figure 2 showed the trend in the overall estimated prevalence of placenta previa in Mainland China. The prevalence was found stable in 2 periods which was from 1971 to 1992 and from 1999 to present, respectively. In the overall trend analysis, the lowest prevalence was 0.31% in 1998 and the highest prevalence was 2.99% in 1993. Generally, the trend in the prevalence of placenta previa in Mainland China from 1960 to present, especially after 2000, was steady.

### 3.8 Geographical distributions of placenta previa

**Figure 3:**
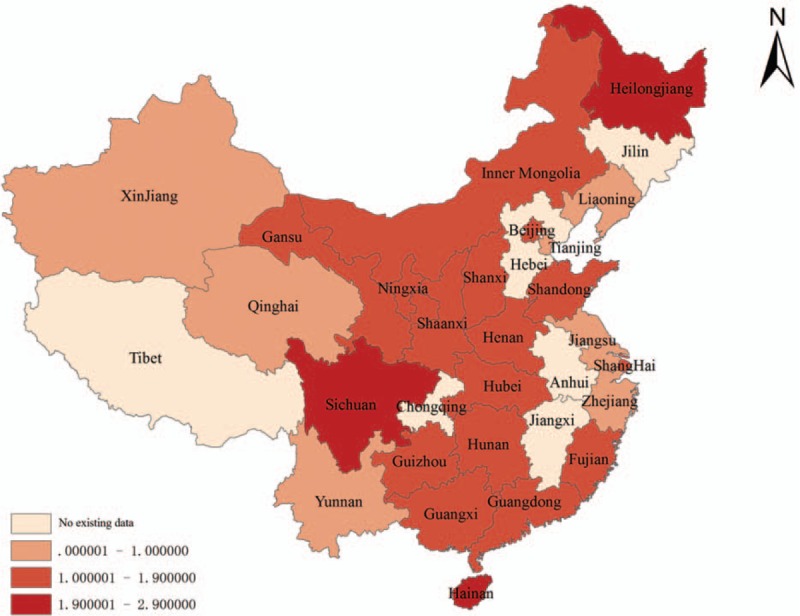
The provincial distribution of the prevalence of placenta previa on map of Mainland China.

Figure 3 showed a color-coded map illustrating the distribution of the prevalence of placenta previa in Mainland China (data available in the following provinces: Liaoning, Heilongjiang, Beijing, Tianjin, Shanxi, Inner Mongolia, Shaanxi, Ningxia, Xinjiang, Qinghai, Gansu, Hunan, Hubei, Henan, Shandong, Jiangsu, Zhejiang, Shanghai, Fujian, Guangdong, Guangxi, Hainan, Sichuan, Yunnan, and Guizhou). The prevalence of placenta previa in the provincial regions of Mainland China ranged from 0.54% in the Liaoning province to 2.90% in Hainan province. We created 4 distribution zones based on the prevalence of placenta previa. The first level represented no available data in the relevant regions (Tibet, Chongqing, Jiangxi, Anhui, and Hebei) and was pink on the map. The highest prevalence of placenta previa, observed in Hainan and more than 5 times the prevalence in Liaoning, belonged to the fourth level, shown on the map in the darkest red. Following the highest prevalence in Hainan (2.90%), the prevalence of placenta previa ranked the second highest in Sichuan (2.48%) and Helongjiang (1.98%), also belonged to the fourth level, then Gansu (1.87%), Guizhou (1.63%), Guangxi (1.49%), Hubei (1.41%), Guangdong (1.26%), Inner Mongolia (1.28%), Fujian (1.20%), Beijing (1.19%), Shanghai (1.16%), Hunan (1.15%), Shaanxi (1.11%), Shandong (1.10%), Ningxia (1.06%), Shanxi (1.06%), and Henan (1.02%), which all belonged to the third level. The second level distribution zone appeared in light red on the map and included Jiangsu (0.93%), Yunnan (0.82%), Xinjiang (0.77%), Zhejiang (0.71%), Tianjin (0.70%), Qinghai (0.66%), and Liaoning (0.54%). Overall, no particular concentration in the distribution of placenta previa prevalence was indicated on the map.

## 4 Discussion

Because of the time-consuming and high-cost of epidemiological survey in the largest population in the world, a national epidemiological survey of placenta previa, which including most of provinces, has never been performed to date in the pregnancies population in a vast territory of Mainland China. The purpose of this study was to explore the overall prevalence of placenta previa in Mainland China from 1965 through 2015 and also to explore the discrepancy exhibited by mean age, survey time, hospital level, and geographic distributions. To the best of our knowledge, this study is the first meta-analysis pooling the prevalence of placenta previa attempts among deliveries in Mainland China. In this meta-analysis, 80 eligible retrospective cohort studies, with a total of 1,298,548 subjects, were included. We found that the pooled overall prevalence of placenta previa among deliveries was 1.24% (95% CI, 1.12–1.36) in Mainland China during 1965 to 2015. The trend in the prevalence of placenta previa in Mainland China was steady. The occurrence rate of placenta previa in the region groups of Northeast, North, Northwest, Central China, East, South, and Southwest was 1.20%, 1.01%, 1.10%, 1.15%, 0.93%, 1.42%, and 2.01%, respectively. The prevalence map indicated that the geographic distributions of placenta previa were unequal in Mainland China. No statistically significant difference was observed in the prevalence of placenta previa in survey year groups, quality score, and hospital level. Through the regression model, we did not find a significant heterogeneity for the potential source of 5 variables listed (region, mean age, publication year, quality score, and hospital level) yet.

The occurrence rates of placenta previa reported in epidemiological studies were considerably different among different countries. Matsuda et al^[15]^ reported in the 125 centers perinatal network in Japan from 2001 through to 2005 study that the prevalence of placenta previa was 1.39 per 100 singleton births, whereas the rate was only 0.42% in a population-based study in Israel.^[2]^ In retrospective studies, the occurrence rates of placenta previa among singleton pregnancies of women were 0.73%, 1.00%, 1.10%, 1.50%, and 2.80% in Saudi Arabia,^[16]^ Greece,^[17]^ Australia,^[18]^ Korea,^[19]^ and USA,^[20]^ respectively. One explanation for the variation in the reported prevalence is due to the geographic or ethnic differences between populations. Evidence has existed that normal gestational length is longer in white European than Black and Asian in nulliparous women with singleton live fetuses at the time of spontaneous labor.^[21]^ Additionally, sample age, socio-economic status, and sampling methods have a profound influence on the prevalence of placenta previa.

The pathophysiology of placenta previa remains largely obscure, but epidemiological and clinical studies have appeared to be an association between endometrial damage and uterine scarring. The clinical risk factors of this disease include advanced maternal age, multiple gestations, previous previa, prior spontaneous or induced abortion, and previous cesarean section.^[22–24]^ It has reported that a cesarean first birth is associated with increased risks of previa and abruption in the second pregnancy, and there is also a dose–response pattern in the risk of previa, with increasing number of prior cesarean deliveries.^[25]^ Furthermore, other behavioral factors for previa include maternal smoking, cocaine, and drug use during pregnancy.^[26]^ A policy has instituted to allow more than one child from a family by the Chinese government in 2015. The incidence of advanced maternal age will increase in the next few years. Furthermore, the cesarean section rate is still much higher than the World Health Organization (WHO) recommendations in Mainland China. Predictably, the incidence of maternal and fetal complications, including placenta previa, will rise further.

The geographic distribution of placenta previa remains unclear in Mainland China. In this study, we used meta-analysis to pool the data from all of the regional surveys on placenta previa in Mainland China from 1965 to 2015, and a prevalence map was constructed using ArcGIS. To some extent, the results may support the planning and implementation of public health policies and may identify future research priorities. Unfortunately, the map only provides information for 25 provincial regions, 6 provinces without data, and we could not find an obvious trend in the geographic distribution of placenta previa. The various occurrence rates of placenta previa in different regions may be partly due to differences in factors such as environmental aspect, economic development, educational status, cultural environments, and medical conditions.

There are several limitations which might affect the outcome should be considered in the meta-analysis study. First, extreme heterogeneity existed in this research. Although the study of the subgroup and meta-regression were all done, it was still high persisted within subgroups based on the variables. In addition, there were several other factors that likely contributed to heterogeneity, including cultural environments, economic status, and medical conditions. However, it was not possible to analyze the effects of these factors because of insufficient data. Meanwhile, publication bias was involved in this research. Second, because of 6 provinces without data, we only obtained data from 25 provinces in Mainland China. And, this may be impact on the results of geographic distribution map. Third, most of the study samples of the included studies were not chosen in random, and there might have been some preference while choosing and confounding, which could not be avoided. Lastly, after half a century of medical development, screening instruments, diagnostic tools, and the criteria for placenta previa have changed. Traditionally, placenta previa is classified as “complete,” “partial,” “marginal,” and “low-lying”. The findings in the few studies have been reported by different groups, and we could not be able to further analysis. Nevertheless, the strength of the present meta-analysis lies in a large sample size (1,298,548 subjects and 14,199 placenta previa cases) from almost all provinces in Mainland China, and our study was generated reasonably precise estimates of the prevalence of placenta previa.

In conclusion, the present meta-analysis explored the prevalence of placenta previa among deliveries in Mainland China. The results showed that the overall prevalence of placenta previs was 1.24%. The trend in the prevalence of placenta previa in Mainland China was steady. And, the prevalence map indicated that the geographic distributions of placenta previa were unequal. This review would be useful for the design of placenta previa planning and implementation adequate health care systems and treatment programs in Mainland China.

## Acknowledgments

The authors appreciate the efforts of all the researchers whose articles were included in this study. Thanks to Bao Liang, from Department of Occupational Health and Environmental Health, School of Public Health, Anhui Medical University, helps us draft the geographical distributions map.

## Supplementary Material

Supplemental Digital Content

## Supplementary Material

Supplemental Digital Content

## Supplementary Material

Supplemental Digital Content

## Supplementary Material

Supplemental Digital Content

## Supplementary Material

Supplemental Digital Content

## Supplementary Material

Supplemental Digital Content

## Supplementary Material

Supplemental Digital Content

## References

[1] Silver RM Abnormal placentation: placenta previa, vasa previa, and placenta accreta. *Obstet Gynecol* 2015; 126:654–668. 26244528 10.1097/AOG.0000000000001005

[2] Rosenberg T Pariente G Sergienko R Critical analysis of risk factors and outcome of placenta previa. *Arch Gynecol Obstet* 2011; 284:47–51. 20652281 10.1007/s00404-010-1598-7

[3] Ananth CV Smulian JC Vintzileos AM The effect of placenta previa on neonatal mortality: a population-based study in the United States, 1989 through 1997. *Am J Obstet Gynecol* 2003; 188:1299–1304. 12748502 10.1067/mob.2003.76

[4] Mastrolia SA Baumfeld Y Loverro G Placenta previa associated with severe bleeding leading to hospitalization and delivery: a retrospective population based cohort study. *J Matern Fetal Neonatal Med* 2016; 29:3467–3471. 26653989 10.3109/14767058.2015.1131264

[5] Cresswell JA Ronsmans C Calvert C Prevalence of placenta praevia by world region: a systematic review and meta-analysis. *Trop Med Int Health* 2013; 18:712–724. 23551357 10.1111/tmi.12100

[6] Guo JD Zhang JY Han XY Clinical analysis of 220 cases of placenta previa. *J Harbin Med Univ* 1965; 1:90–95.

[7] Luo XL Zhang WY Obstetrical disease spectrum in China: an epidemiological study of 111,767 cases in 2011. *Chin Med J (Engl)* 2015; 128:1137–1146. 25947393 10.4103/0366-6999.156076 PMC4831537

[8] Wang TT Liu WF Cong KJ The diagnosis treatment and prognosis of placenta previa. *Beijing Med J* 1994; 16:114–115.

[9] Li DM Clinical analysis of 274 cases of placenta previa. *J Beijing Colle Acu-Moxi Orth-Trau* 1997; 4:38–40.

[10] Stroup DF Berlin JA Morton SC Meta-analysis of observational studies in epidemiology: a proposal for reporting. Meta-analysis Of Observational Studies in Epidemiology (MOOSE) group. *JAMA* 2000; 283:2008–2012. 10789670 10.1001/jama.283.15.2008

[11] Moher D Liberati A Tetzlaff J Group P. Preferred reporting items for systematic reviews and meta-analyses: the PRISMA statement. *Int J Surg* 2010; 8:336–341. 20171303 10.1016/j.ijsu.2010.02.007

[12] von Elm E Altman DG Egger M The strengthening the reporting of observational studies in epidemiology (STROBE) statement: guidelines for reporting observational studies. *Lancet* 2007; 370:1453–1457. 18064739 10.1016/S0140-6736(07)61602-X

[13] Wang W Guo Y Zhang D The prevalence of benign prostatic hyperplasia in mainland China: evidence from epidemiological surveys. *Sci Rep* 2015; 5:13546. 26306721 10.1038/srep13546 PMC4549711

[14] DerSimonian R Laird N Meta-analysis in clinical trials revisited. *Contemp Clin Trials* 2015; 45:139–145. 26343745 10.1016/j.cct.2015.09.002 PMC4639420

[15] Matsuda Y Hayashi K Shiozaki A Comparison of risk factors for placental abruption and placenta previa: case-cohort study. *J Obstet Gynaecol Res* 2011; 37:538–546. 21375675 10.1111/j.1447-0756.2010.01408.x

[16] Bahar A Abusham A Eskandar M Risk factors and pregnancy outcome in different types of placenta previa. *J Obstet Gynaecol Can* 2009; 31:126–131. 19327211 10.1016/s1701-2163(16)34096-8

[17] Daskalakis G Simou M Zacharakis D Impact of placenta previa on obstetric outcome. *Int J Gynaecol Obstet* 2011; 114:238–241. 21704999 10.1016/j.ijgo.2011.03.012

[18] McCormack RA Doherty DA Magann EF Antepartum bleeding of unknown origin in the second half of pregnancy and pregnancy outcomes. *BJOG* 2008; 115:1451–1457. 18715242 10.1111/j.1471-0528.2008.01856.x

[19] Jang DG We JS Shin JU Maternal outcomes according to placental position in placental previa. *Int J Med Sci* 2011; 8:439–444. 21814478 10.7150/ijms.8.439 PMC3149424

[20] Eichelberger KY Haeri S Kessler DC Placenta previa in the second trimester: sonographic and clinical factors associated with its resolution. *Am J Perinatol* 2011; 28:735–739. 21660901 10.1055/s-0031-1280853 PMC3175253

[21] Patel RR Steer P Doyle P Does gestation vary by ethnic group? A London-based study of over 122,000 pregnancies with spontaneous onset of labour. *Int J Epidemiol* 2004; 33:107–113. 15075154 10.1093/ije/dyg238

[22] Hung TH Hsieh CC Hsu JJ Risk factors for placenta previa in an Asian population. *Int J Gynaecol Obstet* 2007; 97:26–30. 17316644 10.1016/j.ijgo.2006.12.006

[23] Downes KL Hinkle SN Sjaarda LA Previous prelabor or intrapartum cesarean delivery and risk of placenta previa. *Am J Obstet Gynecol* 2015; 212:669 e661–669 e666. 25576818 10.1016/j.ajog.2015.01.004 PMC4416991

[24] Kollmann M Gaulhofer J Lang U Placenta praevia: incidence, risk factors and outcome. *J Matern Fetal Neonatal Med* 2016; 29:1395–1398. 26043298 10.3109/14767058.2015.1049152

[25] Getahun D Oyelese Y Salihu HM Previous cesarean delivery and risks of placenta previa and placental abruption. *Obstet Gynecol* 2006; 107:771–778. 16582111 10.1097/01.AOG.0000206182.63788.80

[26] Usta IM Hobeika EM Musa AA Placenta previa-accreta: risk factors and complications. *Am J Obstet Gynecol* 2005; 193:1045–1049. 16157109 10.1016/j.ajog.2005.06.037

