# Supplementary material for: Prevalence of placenta previa among deliveries in Mainland China: A PRISMA-compliant systematic review and meta-analysis
Source: Medicine (Baltimore). 2016 Oct 7;95(40):e5107. doi: 10.1097/MD.0000000000005107 (PMC5059095; doi:10.1097/MD.0000000000005107)
Supplement: Supplemental Digital Content [file medi-95-e5107-s001.doc]

Supplementary Table 1. MOOSE Checklist. (DOC)

**MOOSE Checklist for Meta-analyses of Observational Studies**

| **Item No** | **Recommendation** | **Reported on Page No** |
| --- | --- | --- |
| Reporting of background should include | | |
| 1 | Problem definition | Page 4 |
| 2 | Hypothesis statement | Page 4 |
| 3 | Description of study outcome(s) | Page 4 |
| 4 | Type of exposure or intervention used | Page 4 |
| 5 | Type of study designs used | Page 4 |
| 6 | Study population | Page 4 |
| Reporting of search strategy should include | | |
| 7 | Qualifications of searchers (eg, librarians and investigators) | Page 5 |
| 8 | Search strategy, including time period included in the synthesis and key words | Page 5 |
| 9 | Effort to include all available studies, including contact with authors | Page 5 |
| 10 | Databases and registries searched | Page 5 |
| 11 | Search software used, name and version, including special features used (eg, explosion) | Page 5 |
| 12 | Use of hand searching (eg, reference lists of obtained articles) | Page 5 |
| 13 | List of citations located and those excluded, including justification | Page 5 |
| 14 | Method of addressing articles published in languages other than English | Page 5 |
| 15 | Method of handling abstracts and unpublished studies | Page 5 |
| 16 | Description of any contact with authors | Page 5 |
| Reporting of methods should include | | |
| 17 | Description of relevance or appropriateness of studies assembled for assessing the hypothesis to be tested | Page 6 |
| 18 | Rationale for the selection and coding of data (eg, sound clinical principles or convenience) | Page 6 |
| 19 | Documentation of how data were classified and coded (eg, multiple raters, blinding and interrater reliability) | Page 6 |
| 20 | Assessment of confounding (eg, comparability of cases and controls in studies where appropriate) | Page 6 |
| 21 | Assessment of study quality, including blinding of quality assessors, stratification or regression on possible predictors of study results | Page 6 |
| 22 | Assessment of heterogeneity | Page 6 |
| 23 | Description of statistical methods (eg, complete description of fixed or random effects models, justification of whether the chosen models account for predictors of study results, dose-response models, or cumulative meta-analysis) in sufficient detail to be replicated | Page 6 |
| 24 | Provision of appropriate tables and graphics | Page 6 |
| Reporting of results should include | | |
| 25 | Graphic summarizing individual study estimates and overall estimate | Page 7 |
| 26 | Table giving descriptive information for each study included | Page 7-8 |
| 27 | Results of sensitivity testing (eg, subgroup analysis) | Page 8-9 |
| 28 | Indication of statistical uncertainty of findings | Page 8-9 |

| **Item No** | **Recommendation** | **Reported on Page No** |
| --- | --- | --- |
| Reporting of discussion should include | | |
| 29 | Quantitative assessment of bias (eg, publication bias) | Page 10 |
| 30 | Justification for exclusion (eg, exclusion of non-English language citations) | Page 10 |
| 31 | Assessment of quality of included studies | Page 10 |
| Reporting of conclusions should include | | |
| 32 | Consideration of alternative explanations for observed results | Page 11-12 |
| 33 | Generalization of the conclusions (ie, appropriate for the data presented and within the domain of the literature review) | Page 12 |
| 34 | Guidelines for future research | Page 12 |
| 35 | Disclosure of funding source | Page 12 |

*From*: Stroup DF, Berlin JA, Morton SC, et al, for the Meta-analysis Of Observational Studies in Epidemiology (MOOSE) Group. Meta-analysis of Observational Studies in Epidemiology. A Proposal for Reporting. *JAMA*. 2000;283(15):2008-2012. doi: 10.1001/jama.283.15.2008.

Transcribed from the original paper within the NEUROSURGERY® Editorial Office, Atlanta, GA, United Sates. August 2012.

Supplementary Table 2. PRISMA Checklist. (DOC)

| **Section/topic** | **#** | **Checklist item** | **Reported on page #** |
| --- | --- | --- | --- |
| **TITLE** | | |  |
| Title | 1 | Identify the report as a systematic review, meta-analysis, or both. | 3 |
| **ABSTRACT** | | |  |
| Structured summary | 2 | Provide a structured summary including, as applicable: background; objectives; data sources; study eligibility criteria, participants, and interventions; study appraisal and synthesis methods; results; limitations; conclusions and implications of key findings; systematic review registration number. | 3 |
| **INTRODUCTION** | | |  |
| Rationale | 3 | Describe the rationale for the review in the context of what is already known. | 4 |
| Objectives | 4 | Provide an explicit statement of questions being addressed with reference to participants, interventions, comparisons, outcomes, and study design (PICOS). | 4 |
| **METHODS** | | |  |
| Protocol and registration | 5 | Indicate if a review protocol exists, if and where it can be accessed (e.g., Web address), and, if available, provide registration information including registration number. | 5 |
| Eligibility criteria | 6 | Specify study characteristics (e.g., PICOS, length of follow-up) and report characteristics (e.g., years considered, language, publication status) used as criteria for eligibility, giving rationale. | 5 |
| Information sources | 7 | Describe all information sources (e.g., databases with dates of coverage, contact with study authors to identify additional studies) in the search and date last searched. | 5 |
| Search | 8 | Present full electronic search strategy for at least one database, including any limits used, such that it could be repeated. | 5 |
| Study selection | 9 | State the process for selecting studies (i.e., screening, eligibility, included in systematic review, and, if applicable, included in the meta-analysis). | 5 |
| Data collection process | 10 | Describe method of data extraction from reports (e.g., piloted forms, independently, in duplicate) and any processes for obtaining and confirming data from investigators. | 5-6 |
| Data items | 11 | List and define all variables for which data were sought (e.g., PICOS, funding sources) and any assumptions and simplifications made. | 5 |
| Risk of bias in individual studies | 12 | Describe methods used for assessing risk of bias of individual studies (including specification of whether this was done at the study or outcome level), and how this information is to be used in any data synthesis. | 6 |
| Summary measures | 13 | State the principal summary measures (e.g., risk ratio, difference in means). | 6 |
| Synthesis of results | 14 | Describe the methods of handling data and combining results of studies, if done, including measures of consistency (e.g., I^2^) for each meta-analysis. | 6 |

Page 1 of 2

| **Section/topic** | **#** | **Checklist item** | **Reported on page #** |
| --- | --- | --- | --- |
| Risk of bias across studies | 15 | Specify any assessment of risk of bias that may affect the cumulative evidence (e.g., publication bias, selective reporting within studies). | 6 |
| Additional analyses | 16 | Describe methods of additional analyses (e.g., sensitivity or subgroup analyses, meta-regression), if done, indicating which were pre-specified. | 6 |
| **RESULTS** | | |  |
| Study selection | 17 | Give numbers of studies screened, assessed for eligibility, and included in the review, with reasons for exclusions at each stage, ideally with a flow diagram. | 7, Fig.1. |
| Study characteristics | 18 | For each study, present characteristics for which data were extracted (e.g., study size, PICOS, follow-up period) and provide the citations. | 7 |
| Risk of bias within studies | 19 | Present data on risk of bias of each study and, if available, any outcome level assessment (see item 12). | 7, Sup Table 3 |
| Results of individual studies | 20 | For all outcomes considered (benefits or harms), present, for each study: (a) simple summary data for each intervention group (b) effect estimates and confidence intervals, ideally with a forest plot. | 7-8, Sup Fig. 1-6. |
| Synthesis of results | 21 | Present results of each meta-analysis done, including confidence intervals and measures of consistency. | 7-9 |
| Risk of bias across studies | 22 | Present results of any assessment of risk of bias across studies (see Item 15). | 7-9 |
| Additional analysis | 23 | Give results of additional analyses, if done (e.g., sensitivity or subgroup analyses, meta-regression [see Item 16]). | 9-10 |
| **DISCUSSION** | | |  |
| Summary of evidence | 24 | Summarize the main findings including the strength of evidence for each main outcome; consider their relevance to key groups (e.g., healthcare providers, users, and policy makers). | 10 |
| Limitations | 25 | Discuss limitations at study and outcome level (e.g., risk of bias), and at review-level (e.g., incomplete retrieval of identified research, reporting bias). | 12 |
| Conclusions | 26 | Provide a general interpretation of the results in the context of other evidence, and implications for future research. | 12 |
| **FUNDING** | | |  |
| Funding | 27 | Describe sources of funding for the systematic review and other support (e.g., supply of data); role of funders for the systematic review. | 12 |

*From:*  Moher D, Liberati A, Tetzlaff J, Altman DG, The PRISMA Group (2009). Preferred Reporting Items for Systematic Reviews and Meta-Analyses: The PRISMA Statement. PLoS Med 6(6): e1000097. doi:10.1371/journal.pmed1000097

For more information, visit: **www.prisma-statement.org**.

Page 2 of 2

Supplementary Table 3. Characteristics of the included studies and quality scores for assessing the risk of bias in the individual studies.

| Ref | Author/Year | Region | Province | Age* | Enrolment Period | Sample  size | Case  size | Hospital  Level | Prevalence (%);  [95% CI] | Quality  score |
| --- | --- | --- | --- | --- | --- | --- | --- | --- | --- | --- |
| 1 | Luo et al./2015 | National | Mixed 14 provinces | 18.00-29.00 | 201001-201112 | 111767 | 1367 | Secondary | 1.22[1.16-1.29] | 9 |
| 2 | Li et al./2014 |  |  | 30.43±5.17 | 201101-201112 | 108049 | 1304 | Secondary | 1.21[1.14-1.27] | 9 |
| 2.1 |  | Northeast | Northeast |  |  | 16518 | 184 |  | 1.11[0.95-1.27] |  |
| 2.2 |  | North | North |  |  | 34852 | 287 |  | 0.82[0.73-0.92] |  |
| 2.3 |  | Central China | Central China |  |  | 5541 | 26 |  | 0.47[0.29-0.65] |  |
| 2.4 |  | South | Guangdong |  |  | 21562 | 280 |  | 1.30[1.15-1.45] |  |
| 2.5 |  | Central China | Hubei |  |  | 8828 | 92 |  | 1.04[0.83-1.25] |  |
| 2.6 |  | Southwest | Sichuan |  |  | 9686 | 350 |  | 3.61[3.24-3.99] |  |
| 2.7 |  | Northwest | Xinjiang |  |  | 11062 | 85 |  | 0.77[0.61-0.93] |  |
| 3 | Yang et al./2014 | Southwest | Guizhou | 26.00±4.50 | 200901-201401 | 7186 | 83 | Secondary | 1.16[0.91-1.40] | 6 |
| 4 | Yu et al./2014 | East | Zhejiang | 29.00-34.00 | 201001-201307 | 4565 | 42 | Secondary | 0.92[0.64-1.20] | 7 |
| 5 | Yu et al./2014 | South | Guangdong | 25.00-35.00 | 200912-201212 | 3960 | 60 | Secondary | 1.52[1.13-1.90] | 7 |
| 6 | Fu et al./2014 | North | Beijing | 31.60±5.50 | 200901-201206 | 9086 | 222 | Tertiary | 2.44[2.13-2.76] | 8 |
| 7 | Li et al./2014 | North | Inner Mongolia | 30.00±4.80 | 201101-201306 | 16578 | 252 | Tertiary | 1.52[1.33-1.71] | 6 |
| 8 | Chen et al./2013 | Southwest | Sichuan | 28.6±5.42 | 200901-201012 | 34014 | 1432 | Secondary | 4.21[4.00-4.42] | 8 |
| 9 | Bai et al./2013 | Central China | Henan | --- | 200601-201010 | 13464 | 219 | Secondary | 1.63[1.41-1.84] | 6 |
| 10 | Li et al./2013 | Northwest | Gansu | 30.68±5.16 | 200805-201201 | 10576 | 198 | Tertiary | 1.87[1.61-2.13] | 7 |
| 11 | Li et al./2013 | South | Guangdong | --- | 201101-201112 | 9385 | 117 | Secondary | 1.25[1.02-1.47] | 8 |
| 12 | Zhao et al./2013 | North | Shanxi | 20.00-39.00 | 201001-201206 | 9056 | 102 | Secondary | 1.13[0.91-1.34] | 6 |
| 13 | Feng et al./2013 | Central China | Henan | --- | 200901-201101 | 2380 | 26 | Secondary | 1.09[0.67-1.51] | 5 |
| 14 | Cheng et al./2013 | Southwest | Guizhou | 24.80±5.80 | 200802-201302 | 986 | 14 | Secondary | 1.42[0.68-2.16] | 6 |
| 15 | Li et al./2012 | Southwest | Sichuan | --- | 200710-201109 | 19218 | 183 | Secondary | 0.95[0.81-1.09] | 7 |
| 16 | Liu et al./2012 | Southwest | Sichuan | 28.20±3.20 | 200104-201108 | 10157 | 221 | Secondary | 2.18[1.89-2.46] | 6 |
| 17 | Yan et al./2012 | East | Jiangsu | 20.00-41.00 | 200801-201112 | 9825 | 55 | Secondary | 0.56[0.41-0.71] | 6 |
| 18 | Han et al./2012 | South | Guangdong | 22.00-43.00 | 200501-201205 | 4892 | 63 | Secondary | 1.29[0.97-1.60] | 7 |
| 19 | Gu et al./2012 | South | Guangdong | 25.10±4.80 | 200802-201111 | 4010 | 62 | Secondary | 1.55[1.16-1.93] | 7 |
| 20 | Chen et al./2012 | South | Guangdong | 25.60±5.40 | 200802-201111 | 3865 | 61 | Secondary | 1.58[1.19-1.97] | 7 |
| 21 | Zhang et al./2012 | Northwest | Shaanxi | 30.64±5.10 | 200901-201112 | 3333 | 50 | Tertiary | 1.50[1.09-1.91] | 6 |
| 22 | Zhou et al./2012 | South | Guangxi | 22.00-40.00 | 200801-201112 | 3165 | 58 | Secondary | 1.83[1.37-2.30] | 7 |
| 23 | Liu et al./2011 | Central China | Hunan | 28.40±4.50 | 200806-201105 | 24213 | 371 | Tertiary | 1.53[1.38-1.69] | 6 |
| 24 | Du et al./2011 | North | Tianjin | 30.50±5.10 | 200001-201007 | 16330 | 110 | Secondary | 0.67[0.55-0.80] | 6 |
| 25 | Zhang et al./2011 | South | Hainan | 21.00-39.00 | 200001-200912 | 5568 | 46 | Tertiary | 0.83[0.59-1.06] | 5 |
| 26 | Wang et al./2011 | North | Inner Mongolia | 20.00-42.00 | 200601-200912 | 5326 | 54 | Secondary | 1.01[0.74-1.28] | 5 |
| 27 | Wu et al./2010 | South | Fujian | 17.00-43.00 | 200401-200812 | 18741 | 225 | Secondary | 1.20[1.04-1.36] | 6 |
| 28 | Jing et al./2010 | Southwest | Sichuan | 16.00-50.00 | 200001-200812 | 9850 | 145 | Secondary | 1.47[1.23-1.71] | 6 |
| 29 | Wei et al./2010 | Northwest | Shaanxi | 18.00-40.00 | 200501-200801 | 4810 | 47 | Secondary | 0.98[0.70-1.26] | 5 |
| 30 | Zhang et al./2010 | Northwest | Ningxia | 31.20±3.50 | 200401-200905 | 3774 | 40 | Secondary | 1.06[0.73-1.39] | 5 |
| 31 | Sun et al./2009 | East | Jiangsu | 22.00-41.00 | 200101-200412 | 10834 | 82 | Secondary | 0.76[0.59-0.92] | 5 |
| 32 | Han et al./2009 | North | Tianjin | 19.00-42.00 | 200001-200712 | 20128 | 104 | Secondary | 0.52[0.42-0.62] | 6 |
| 33 | Wang et al./2008 | East | Shanghai | 30.00±5.00 | 200201-200806 | 36669 | 427 | Secondary | 1.16[1.05-1.27] | 8 |
| 34 | Zheng et al./2008 | Central China | Hunan | 27.81±4.89 | 200006-200706 | 12834 | 86 | Tertiary | 0.67[0.53-0.81] | 7 |
| 35 | Sun et al./2008 | North | Beijing | 22.00-42.00 | 200201-200701 | 11320 | 106 | Secondary | 0.94[0.76-1.11] | 5 |
| 36 | Tang et al./2008 | South | Guangdong | 20.00-43.00 | 200201-200801 | 8060 | 92 | Secondary | 1.14[0.91-1.37] | 5 |
| 37 | Zhu et al./2008 | East | Jiangsu | 27.50±6.37 | 200501-200712 | 6530 | 68 | Secondary | 1.04[0.80-1.29] | 5 |
| 38 | Xiao et al./2007 | Central China | Hunan | 26.90±4.20 | 199901-200612 | 4628 | 72 | Secondary | 1.56[1.20-1.91] | 7 |
| 39 | Wei et al./2007 | South | Guangdong | 18.00-43.00 | 200001-200701 | 3016 | 46 | Secondary | 1.53[1.09-1.96] | 5 |
| 40 | Kong et al./2007 | South | Guangdong | 25.00-36.00 | 200601-200612 | 1496 | 23 | Tertiary | 1.54[0.91-2.16] | 5 |
| 41 | Hong et al./2006 | South | Guangdong | 22.00-38.00 | 199601-200401 | 16320 | 126 | Secondary | 0.77[0.64-0.91] | 6 |
| 42 | Wu et al./2006 | South | Guangdong | 20.00-38.00 | 200201-200512 | 6140 | 143 | Secondary | 2.33[1.95-2.71] | 6 |
| 43 | Jiang et al./2003 | Northeast | Liaoning | --- | 199301-200112 | 8357 | 26 | Secondary | 0.31[0.19-0.43] | 5 |
| 44 | Cheng et al./2003 | National | Mixed 3provinces | 25.75±2.54 | 199808-200102 | 14071 | 122 | Secondary | 0.87[0.71-1.02] | 9 |
| 45 | Liu et al./2002 | North | Tianjin | 22.00-41.00 | 199101-200012 | 13982 | 129 | Secondary | 0.92[0.76-1.08] | 4 |
| 46 | Zhang et al./2001 | Central China | Hubei | --- | 199101-200012 | 6782 | 219 | Secondary | 3.23[2.81-3.65] | 5 |
| 47 | Wang et al./1999 | Central China | Hubei | 17.00-43.00 | 199101-199805 | 33758 | 168 | Tertiary | 0.50[0.42-0.57] | 4 |
| 48 | He et al./1999 | East | Zhejiang | 26.78±3.97 | 197601-199808 | 26489 | 182 | Secondary | 0.69[0.59-0.79] | 6 |
| 49 | Feng et al./1999 | East | Shandong | 19.00-44.00 | 198801-199701 | 25640 | 258 | Secondary | 1.01[0.88-1.13] | 5 |
| 50 | Xie et al./1999 | East | Zhejiang | 23.00-32.00 | 198501-199412 | 7443 | 68 | Secondary | 0.91[0.70-1.13] | 4 |
| 51 | Hao et al./1998 | North | Shanxi | 22.00-30.00 | 198201-199301 | 16746 | 173 | Tertiary | 1.03[0.88-1.19] | 5 |
| 52 | Sheng et al./1998 | Northeast | Heilongjiang | --- | 198701-199601 | 4212 | 80 | Secondary | 1.90[1.49-2.31] | 4 |
| 53 | Yu et al./1998 | East | Jiangsu | 21.00-28.00 | 199201-199601 | 3860 | 56 | Secondary | 1.45[1.07-1.83] | 5 |
| 54 | Li et al./1998 | South | Guangxi | 19.00-49.00 | 198401-199412 | 28093 | 319 | Secondary | 1.14[1.01-1.26] | 6 |
| 55 | Ye et al./1997 | East | Zhejiang | 26.33±3.11 | 198101-199412 | 18109 | 76 | Secondary | 0.42[0.33-0.51] | 5 |
| 56 | Lei et al./1997 | Northwest | Qinghai | 28.24±4.35 | 197601-199604 | 16667 | 110 | Tertiary | 0.66[0.54-0.78] | 5 |
| 57 | Li et al./1997 | South | Hainan | 26.80±4.50 | 199206-199506 | 5482 | 274 | Secondary | 5.00[4.42-5.58] | 5 |
| 58 | Zhang et al./1997 | East | Shanghai | 29.00±5.10 | 199208-199601 | 2910 | 32 | Tertiary | 1.10[0.72-1.48] | 6 |
| 59 | Wang et al./1996 | Central China | Henan | 20.00-30.00 | 198501-199402 | 11215 | 84 | Tertiary | 0.75[0.59-0.91] | 6 |
| 60 | Li et al./1995 | Central China | Hubei | 21.00-35.00 | 198001-199001 | 18945 | 360 | Secondary | 1.90[1.71-2.09] | 5 |
| 61 | Tang et al./1995 | South | Guangxi | 18.00-40.00 | 199001-199401 | 2854 | 46 | Secondary | 1.61[1.15-2.07] | 6 |
| 62 | Li et al./1994 | South | Guangdong | 26.00-35.00 | 198306-199206 | 37012 | 128 | Secondary | 0.35[0.29-0.41] | 6 |
| 63 | Wu et al./1994 | South | Guangdong | 21.00-35.00 | 198301-199101 | 14052 | 137 | Secondary | 0.97[0.81-1.14] | 4 |
| 64 | Cao et al./1994 | South | Guangdong | 21.00-42.00 | 199101-199201 | 4436 | 36 | Secondary | 0.81[0.55-1.08] | 5 |
| 65 | Kan et al./1994 | South | Guangdong | 20.00-30.00 | 198707-199012 | 3050 | 28 | Secondary | 0.92[0.58-1.26] | 5 |
| 66 | Wang et al./1994 | North | Beijing | --- | 196001-198912 | 139271 | 332 | Tertiary | 0.24[0.21-0.26] | 6 |
| 67 | Chen et al./1993 | East | Jiangsu | 27.17±5.00 | 198101-199012 | 16336 | 184 | Tertiary | 1.13[0.96-1.29] | 6 |
| 68 | Mo et al./1993 | Central China | Hunan | 22.00-30.00 | 198101-199101 | 14314 | 128 | Secondary | 0.89[0.74-1.05] | 5 |
| 69 | Zhang et al./1993 | East | Jiangsu | 19.00-42.00 | 198701-199012 | 12280 | 136 | Secondary | 1.11[0.92-1.29] | 6 |
| 70 | Li et al./1993 | East | Jiangsu | 20.00-39.00 | 198701-199012 | 11045 | 104 | Secondary | 0.94[0.76-1.12] | 4 |
| 71 | Zhang et al./1992 | Central China | Hubei | 27.74±3.97 | 198101-199012 | 35247 | 208 | Tertiary | 0.59[0.51-0.67] | 5 |
| 72 | Li et al./1992 | Northeast | Liaoning | --- | 199201-199212 | 14131 | 109 | Secondary | 0.77[0.63-0.92] | 4 |
| 73 | Peng et al./1992 | Northwest | Shaanxi | 22.00-43.00 | 198101-199012 | 11500 | 116 | Tertiary | 1.01[0.83-1.19] | 5 |
| 74 | Tang et al./1991 | East | Shandong | --- | 198704-199004 | 7383 | 92 | Secondary | 1.25[0.99-1.50] | 5 |
| 75 | Wu et al./1990 | East | Jiangsu | --- | 198001-198908 | 10417 | 67 | Tertiary | 0.64[0.49-0.80] | 4 |
| 76 | Jiao et al./1989 | Southwest | Yunnan | 21.00-40.00 | 198101-198512 | 7585 | 62 | Tertiary | 0.82[0.61-1.02] | 5 |
| 77 | Zhang et al./1988 | Central China | Henan | 27.24±4.15 | 196601-198307 | 25257 | 162 | Tertiary | 0.64[0.54-0.74] | 6 |
| 78 | Wang et al./1982 | Southwest | Guizhou | 19.00-30.00 | 197101-198012 | 7030 | 161 | Tertiary | 2.29[1.94-2.64] | 5 |
| 79 | Cai et al./1980 | South | Guangdong | --- | 196401-197712 | 25600 | 403 | Tertiary | 1.57[1.42-1.73] | 4 |
| 80 | Guo et al./1965 | Northeast | Heilongjiang | 26.00-30.00 | 195601-196403 | 10919 | 220 | Tertiary | 2.01[1.75-2.28] | 5 |

*Age was shown in mean ± standard deviation or minimum – maximum.

**Reference**

1. Luo XL, Zhang WY. Obstetrical disease spectrum in China: an epidemiological study of 111,767 cases in 2011. *Chin Med J (Engl)* 2015,**128**:1137-1146.

2. Li Q, Wang ZJ, Yu YH, Guo SQ, Guo XL, Xia YY*, et al.* Epidemiological survey on placenta previa in seven regions of China. *Chin J Prac Gynecol & Obste* 2014,**30**:786-790.

3. Yang XM. Clinical analysis of 83 cases of placenta previa. *Guide Chin Med* 2014,**12**:121-123.

4. Yu LQ. The risk factors of placenta previa: a case-control study. *Chin. J of PHM* 2014,**30**:292-293.

5. Yu YT. Analysis of risk factors associated with placenta previa and its effect on pregnancy. *China Prac Med* 2014,**9**:7-9.

6. Fu CW, Liu YT, Yang JQ, Zhou L, Bian XM, Gao JS*, et al.* Placenta previa and postpartum hemorrhage: case analysis of 222 patients with placenta previa. *J Reprod Med* 2014,**23**:224-228.

7. Li T, Yang XY, Liu AJ, Zhu XX. Clinical analysis of 252 cases of complete placenta previa. *Inner Mongolia Med J* 2014,**46**:340-342.

8. Chen M, Zhang L, Wei Q, Fu X, Gao Q, Liu X. Peripartum hysterectomy between 2009 and 2010 in Sichuan, China. *Int J Gynaecol Obstet* 2013, **2:**183-186.

9. Bai JE. Etiology analysis and nursing experience of placenta previa. *J Henan Med College Staff & Workers* 2013,**25**:728-729.

10. Li FP, Tuo SM, Zhao YH. Analysis of placenta previa risk factors. *J Lanzhou Univer (Med Sci)* 2013,**39**:22-24.

11. Li Q, Yu YH, Wang ZJ, Guo SQ, Xia YY, Guo XL*, et al.* The effect of placenta previa, implantation on maternal and neonatal outcomes. *Prog Obstet Gynecol* 2013,**22**:738-740.

12. Zhao ZP. Analysis of 102 cases of placenta previa. *Shanxi Med J* 2013,**42**:311-312.

13. Feng WL. Clinical analysis of 26 placenta previa cases with cesarean section. *Chin Commu Doc* 2013,**15**:40.

14. Cheng H. The relationship between placental adhesion, placenta previa, placenta implantation and artificial abortion. *China & Foreign Med Treat* 2013:106-107.

15. Li YL. The role of the pregnancy management of placenta praevia in the pregnancy outcome. *China Modern Doc* 2012,**50**:155-156.

16. Liu ZH. The clinical analysis of 20 cases placenta previa combined with placenta accrete. *Guide Chin Med* 2012,**10**:363-364.

17. Yan YH, Zhang YX. Clinical analysis of 55 cases of placenta previa. *China Prac Med* 2012,**7**:91-92.

18. Han Y, Zhao CW. Study on influence of placenta previa morbidity factors and its effect on pregnancy outcome. *China Modern Med* 2012,**19**:53-54.

19. Guo DT. Relation of placenta previa, placenta adhesion and placenta implantation with artificial abortion. *China Modern Med* 2012,**19**:166-167.

20. Chen HY. The association between placenta previa, placenta adhesion, placenta implantation and artificial abortion. *Chin & Fore Med Res* 2012,**10**:124-125.

21. Zhang HL, Li LC, Zhang J. Analysis of risk factors and prognosis of placenta previa. *Chin J Aesth Med* 2012,**21**:56-57.

22. Zhou YH, Huang YJ. The analysis between prenatal check condition and pregnancy outcomes in Pingguo, Guangxi. *Guangxi Med J* 2012,**34**:1081-1084.

23. Liu YF. Clinical analysis of 35 cases of placenta previa combined with placenta accreta. *China Mode Med* 2011,**18**:41-42.

24. Du J. Clinical analysis of 110 cases of placenta previa. *J Med Theor & Prac* 2011,**24**:813-814.

25. Zhang LJ, Wei T, Huang C. The association between placenta previa and abortion: a 46 cases study. *J Third Mil Med Univ* 2011,**33**:1425.

26. Wang J. Clinical analysis of 54 cases of placenta previa. *J North China Coal Med Univer* 2011,**13**:73-74.

27. Wu XY, Ding HM. Pathogeny analysis of 225 cases of placenta previa. *Med Information* 2010:553-554.

28. Jing ZY. Clinical Analysis of 145 Cases of placenta praevia. *Med Information* 2010:755-756.

29. Wei MX. Treatment of pregnancy complicated with placenta previa. *Med Forum* 2010,**14**:118-119.

30. Zhang HY, Zhou XY. Clinical analysis of 40 cases of placenta previa. *Ning Xia Med J* 2010,**32**:1220.

31. Rui S. Clinical analysis of 82 cases of placenta previa. *Contem Med* 2009,**15**:38.

32. Han YX. Clinical analysis of 104 cases of placenta previa. *Capital Med* 2009,**10**:37.

33. Wang AY, Huang Y. The relationship between placenta prvia with obstetric risk factor: a report of 427 cases. *Chongqing Med* 2008,**37**:2310-2312.

34. Zheng AJ. Affection of placenta previa on the perineonate. *Chin J Woman & Child Health Res* 2008,**19**:331-332.

35. Sun LY. Analysis of 106 cases of placenta previa and 7 case of placenta accrete. *Med J Chin Peo Heal* 2008,**20**:1549-1550.

36. Tang L, Li X. Clinical analysis of 11 cases of placenta implantation. *J Gannan Med Univer* 2008,**28**:538-539.

37. Zhu S. Clinical analysis of 68 cases of placenta previa. *Jouranl of Modern Medicine & Health* 2008,**24**:1163-1164.

38. Xiao ZS, Xie JP. Relation of Placenta Previa, Placenta Adhesion and Placenta Implantation With Artificial Abortion. *Prac Preve Med* 2007,**14**:1831-1832.

39. Wei LH. Clinical analysis of 46 cases of placenta previa. *CHINA MED HERA* 2007,**4**:149-150.

40. Kong X, Zhang JH, Li Q, Huang XX. Clinical analysis of 5 cases of placenta previa and implantation *J Prac Med* 2007,**23**:3593-3594.

41. Hong XQ. Clincal Analysis of 126 Cases of Placenta Previa and 3 Cases of Placenta Accrete. *J Trop Med* 2005,**6**:444-445.

42. Wu FM, Zhao LY, Gao LJ. Clinical analysis of 143 cases of placenta previa. *CHINA MED HERA* 2006,**3**:37-39.

43. Jiang M, Tao YJ. Analysis to parturition manier of 26 placenta previas. *J Dalian Med Uinver* 2003,**25**:200-201.

44. Cheng YM, Yuan W, Cai WD, Zhang WM, Wang TY, Wang Y*, et al.* Study on the occurence of cesarean section (CS) and factors related to CS in China. *Chin J Epidemiol* 2003,**10**:893-896.

45. Liu ZP. Clinical analysis of 129 cases of placenta previa. *Chin J Urban& Rural Enter Hyg* 2002:29-31.

46. Zhang LF. Research into the Incurrence Rate and Prognosis of Placenta Praevia. *J Wuhan Uni. of Sci. & Tech. (Natural Science Edition)* 2001,**24**:425-426.

47. Wang JF. Nursing care of patients in placenta previa with postpartum hemorrhage *J Nurs Sci* 1999,**14**:159.

48. He CL. Clinical analysis of 182 cases of placenta previa. *Clini Med* 1999,**19**:28-29.

49. Feng FZ, Zhang LH, Fang SL. The effect of magnesium sulfate in treatment of placenta previa. *J Med Theory & Prac* 1999,**12**:97-98.

50. Xie RH. The association between placenta previa and maternal, infant prognosis. *Zhejiang Clini Med J* 1999,**1**:185-186.

51. Hao XY, Wang XH. The effect of placenta previa in the mother and infant. *J DATONG MED COLLEGE* 1998,**18**:16-18.

52. Sheng XB, Wang HQ, Zhang ZQ. The risk factors of vaginal bleeding in late pregnancy. *Chin Prim Health Care* 1998,**12**:32.

53. Yu WP. The effect of expectant treatment in placenta previa. *Med J Communi* 1998,**12**:201.

54. Li MY. Clinical analysis of 319 cases of placenta previa. *J Youjiang Med Colle Nat Minor* 1998,**20**:54-55.

55. Ye LL. Clinical analysis of 47 cases of placenta previa. *HENAN MED INFOR* 1997,**5**:30-31.

56. Lei YX. Clinical analysis of 110 cases of placenta previa. *J Prac Med Tech* 1997,**4**:234-235.

57. Li DM. Clinical analysis of 274 cases of placenta previa. *J Beijing Colle Acup-Moxi & Orth-Trauma* 1997,**4**:38-40.

58. Zhang GH, Zhang DL. Clinical analysis of 32 cases of placenta previa. *J Shanghai Tiedao Univ (Med Sci)* 1997,**11**:42-43.

59. Wang AY, Wang CP, Ma N. Clinical analysis of 84 cases of placenta previa. *CENT PLAINS MED J* 1996,**23**:11-12.

60. Li JR. The delivery mode in placenta previa. *Wuhan Med J* 1995,**19**:173-174.

61. Tang FX. Clinical analysis of 46 cases of placenta previa. *J Guilin Med Colle* 1995,**8**:410-412.

62. Li LW. Clinical analysis of 128 cases of placenta previa. *J Prac Med* 1994,**10**:723.

63. Wu WL. Analysis of placenta previa in 137 cases. *Guangdong Med J* 1994,**15**:99-101.

64. Cao Y. Clinical analysis of 36 cases of placenta previa. *J LANZHOU MED COLLE* 1994,**20**:271.

65. Kan M, Wang YY. Clinical analysis of 28 cases of placenta previa. *MED J LIAONING* 1994,**8**:161.

66. Wang TT, Liu WF, Cong KJ. The diagnosis treatment and prognosis of placenta previa. *BEIJING MED J* 1994,**16**:114-115.

67. Chen XL, Shen YJ. Clinical analysis of 184 cases of placenta previa. *J Nanjing Railway Med Colle* 1993,**12**:33-35.

68. Mo DY, Jia Q, Zhang FJ. Clinical analysis of 128 cases of placenta previa. *Hunan Med J* 1993,**11**:62-63.

69. Zhang XZ, Yang TR. Clinical analysis of 126 cases of placenta previa. *Jiangsu Med J* 1993,**19**:395.

70. Li P, Zhang ZX, Qian S. Clinical analysis of 104 cases of placenta previa. *Railway Med J* 1993,**21**:156-157.

71. Zhang YF, Li WJ. A Clinical Analysis of 109 Cases of Postpartum Hemorrhage Induced by Placenta Previa. *J Tongji Med Univer* 1992,**21**:335-337.

72. Li XX. Diagnosis and treatment of late pregnancy bleeding. *MED J LIAONING* 1992,**6**:129-130.

73. Peng JJ, Huang F, Yang HG, Zhang XL. The prevalence rate of placenta previa in perinatal period. *Chin J Birth Heal & Here* 1992,**68**:68-71.

74. Tang NF, Song XX. The association between placenta previa and artificial abortion. *J JINING MED COLLE* 1991,**14**:46-48.

75. Wu XY. The treatment of placenta previa. *Journal of Medical Postgraduates* 1990,**3**:128-129.

76. Jiao SY, Xiao H, Li M. Clinical analysis of 62 cases of placenta previa. *Med Pharm Yunnan* 1989:170-171.

77. Zhang SJ, Sun YZ, Zhu XQ. Clinical analysis of 162 cases of placenta previa. *Chin J Prac Gyneco & Obste* 1988,**4**:27.

78. Wang JY. Clinical analysis of 116 cases of placenta previa. *Guizhou Med J* 1992:39.

79. Cai SY. Placenta Praevia - Analysis of 403 Cases. *ACTA ACADEMIAE MED ZHONG SHAN* 1980,**1**:312-316.

80. Guo JD, Zhang JY, Han XY. Clinical analysis of 220 cases of placenta previa. *J Harbin Med Univer* 1965:90-95.
