# Supplementary material for: Prevalence of placenta previa among deliveries in Mainland China: A PRISMA-compliant systematic review and meta-analysis
Source: Medicine (Baltimore). 2016 Oct 7;95(40):e5107. doi: 10.1097/MD.0000000000005107 (PMC5059095; doi:10.1097/MD.0000000000005107)

Supplementary Figure 1. Forest plot for the overall estimate of the prevalence of placenta previa.


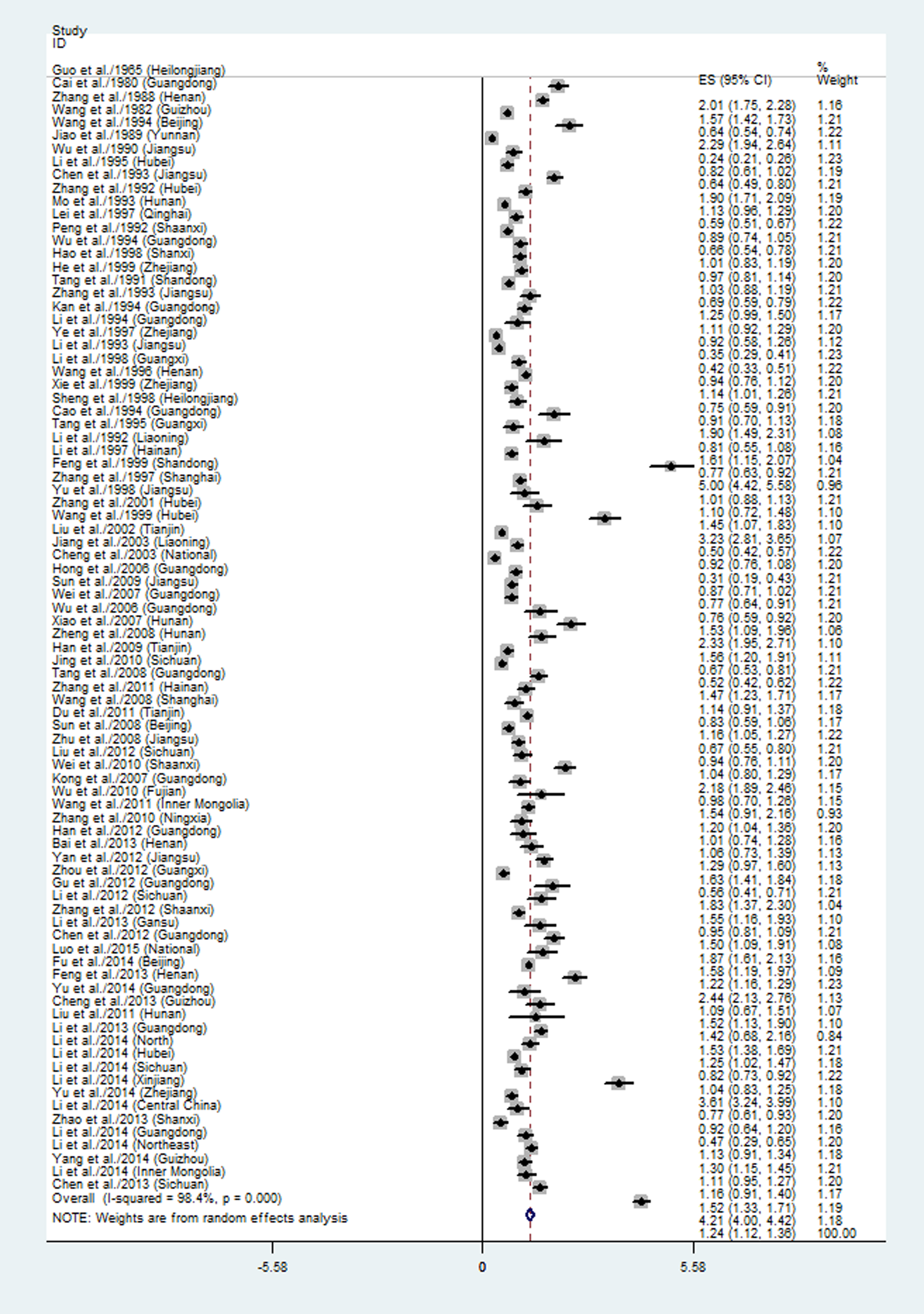

Supplement: Supplemental Digital Content [file medi-95-e5107-s002.doc]
