# Supplementary figures and images for: Prevalence of placenta previa among deliveries in Mainland China: A PRISMA-compliant systematic review and meta-analysis
Source: Medicine (Baltimore). 2016 Oct 7;95(40):e5107. doi: 10.1097/MD.0000000000005107 (PMC5059095; doi:10.1097/MD.0000000000005107)

Supplementary Figure 2. Prevalence of placenta previa by different region groups.

×
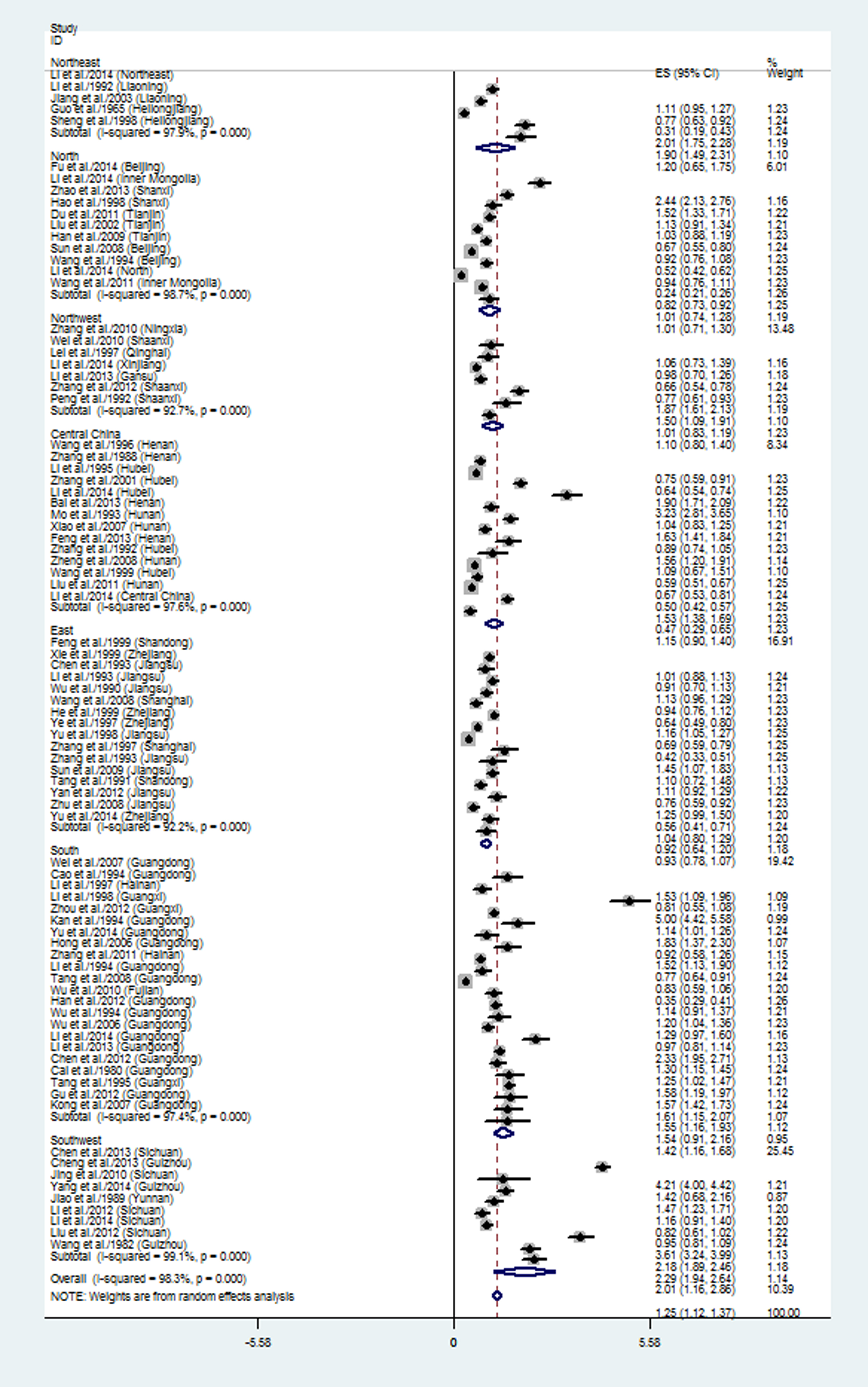

Supplement: Supplemental Digital Content [file medi-95-e5107-s003.doc]

Supplementary Figure 3. Prevalence of placenta previa by different age groups.


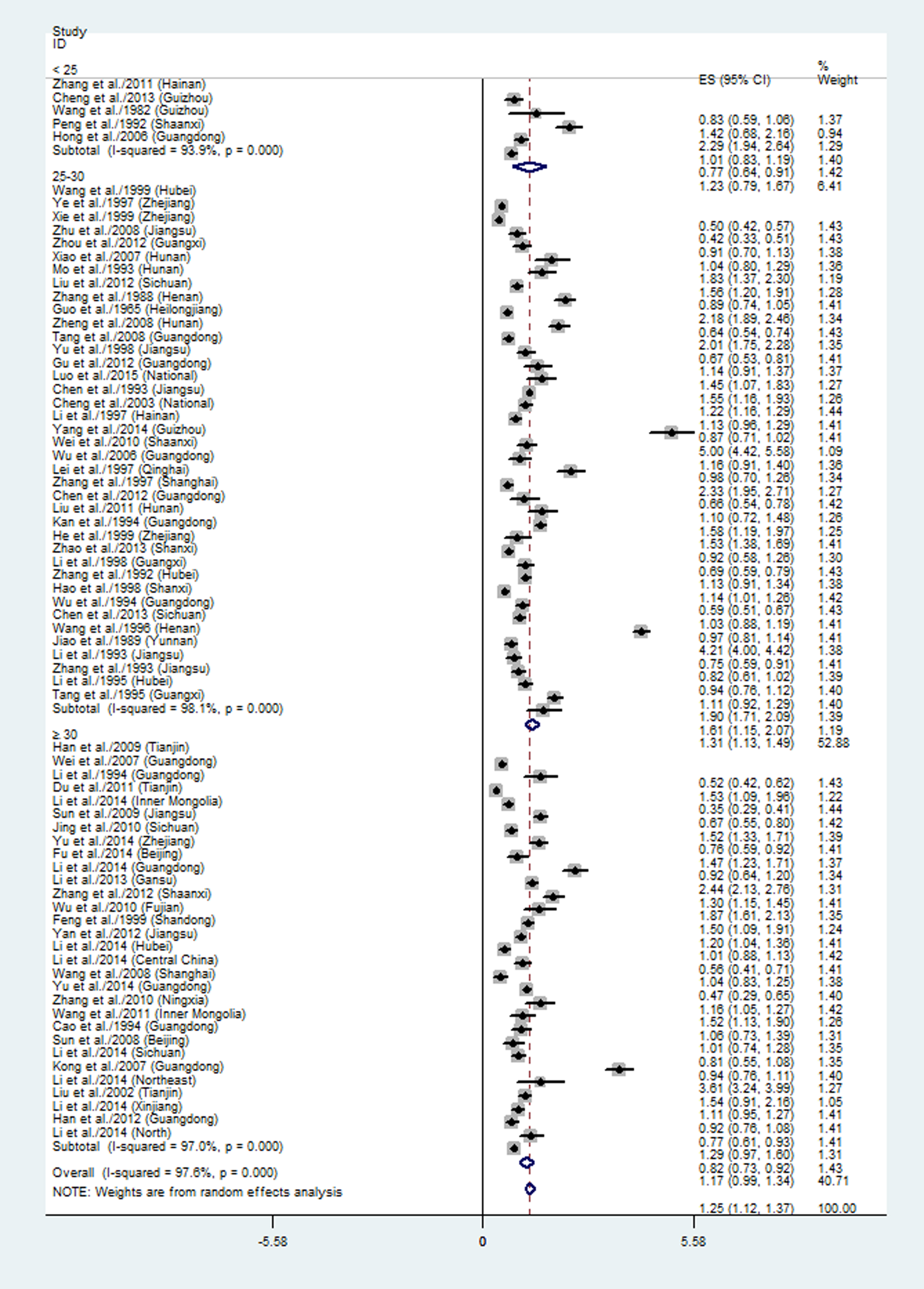

Supplement: Supplemental Digital Content [file medi-95-e5107-s004.doc]

Supplementary Figure 4. Prevalence of placenta previa by different survey year groups.


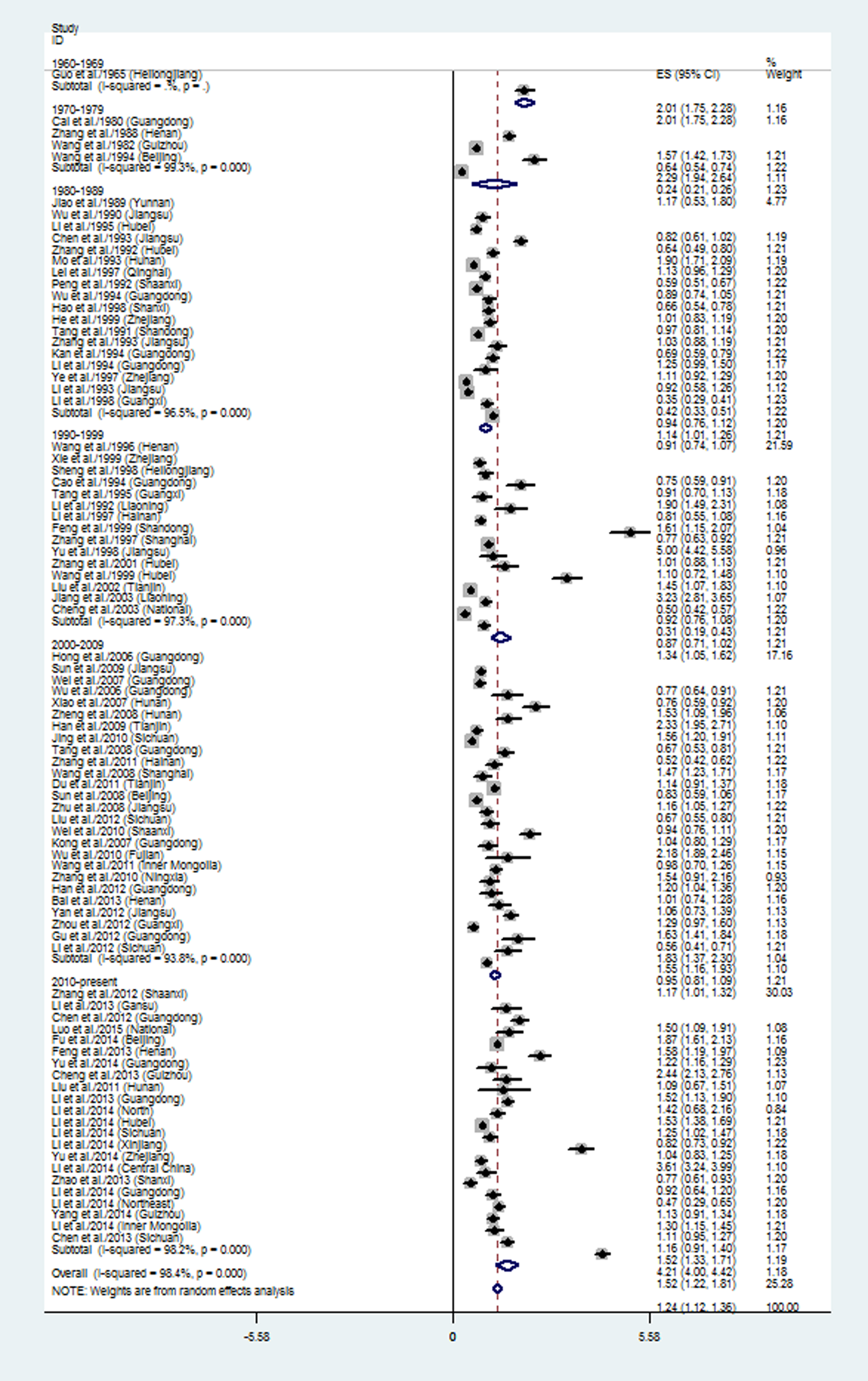

Supplement: Supplemental Digital Content [file medi-95-e5107-s005.doc]

Supplementary Figure 5. Prevalence of placenta previa by different quality score groups.


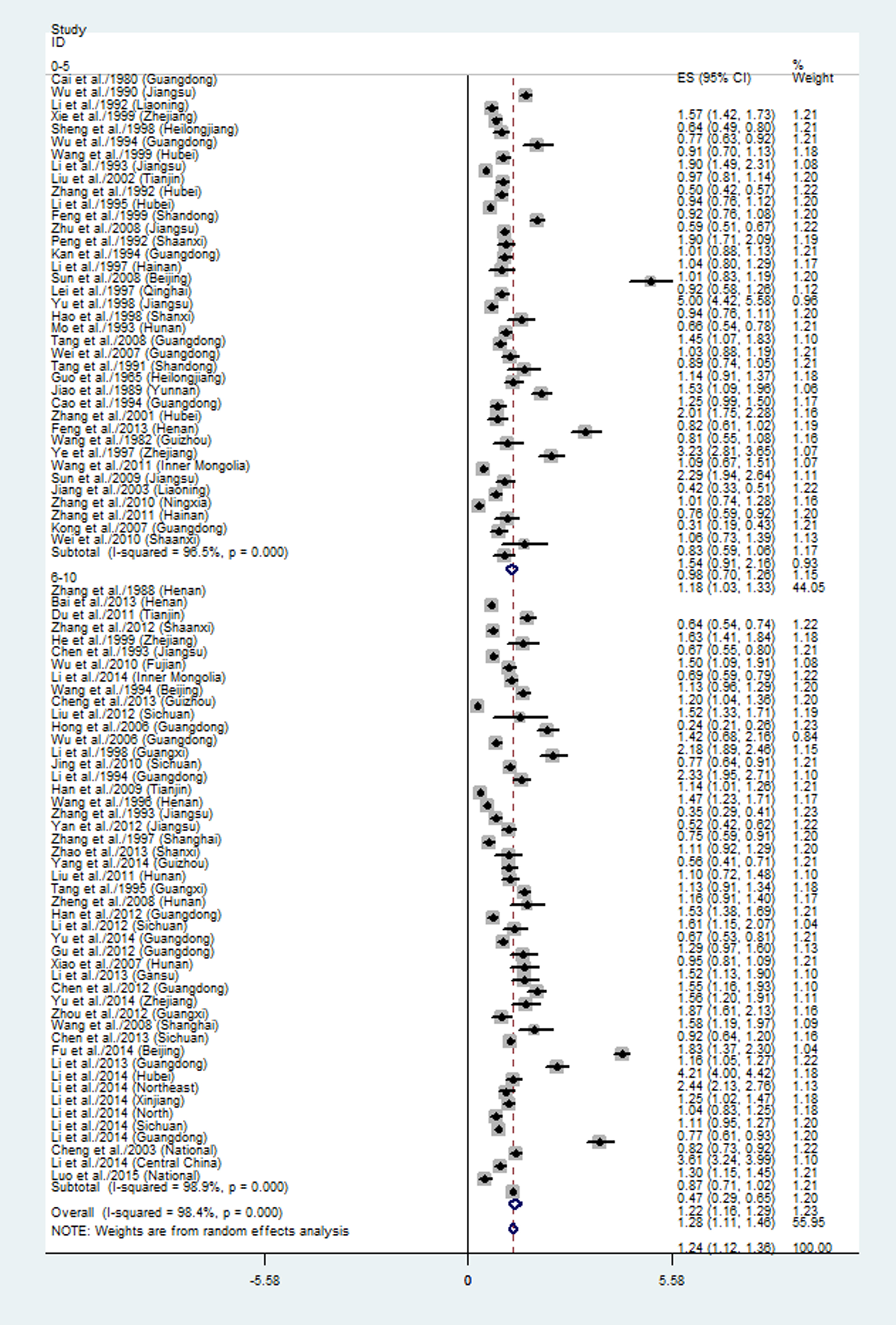

Supplement: Supplemental Digital Content [file medi-95-e5107-s006.doc]

Supplementary Figure 6. Prevalence of placenta previa by different hospital level groups.


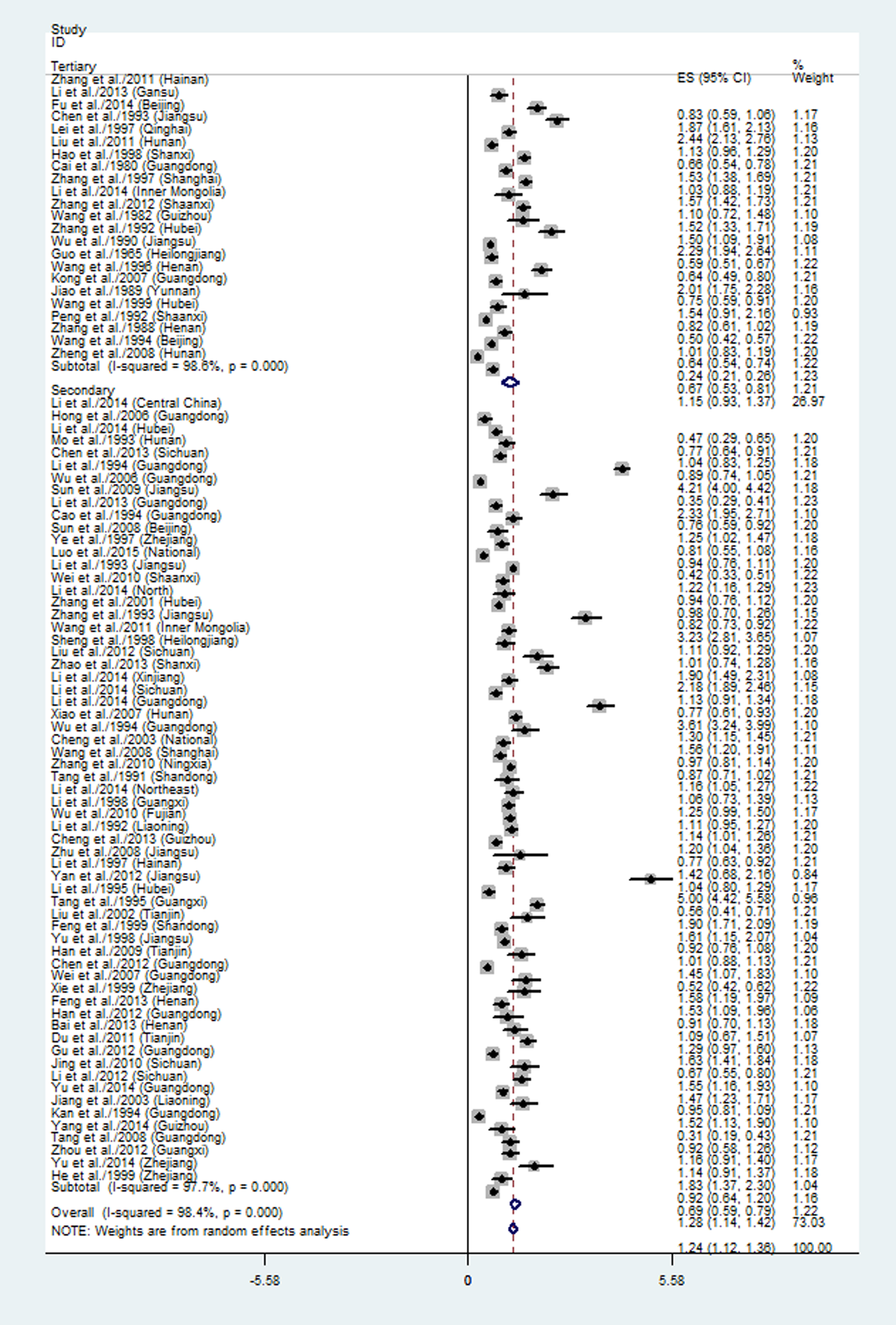

Supplement: Supplemental Digital Content [file medi-95-e5107-s007.doc]
